# Feature selection and validated predictive performance in the domain of *Legionella pneumophila*: a comparative study

**DOI:** 10.1186/s13104-016-1945-2

**Published:** 2016-03-08

**Authors:** Tjeerd van der Ploeg, Ewout W. Steyerberg

**Affiliations:** Department of Science, Medical Center Alkmaar/Inholland University, Alkmaar, The Netherlands; Department of Public Health, Erasmus MC–University Medical Center Rotterdam, Rotterdam, The Netherlands

## Abstract

**Background:**

Genetic comparisons of clinical and environmental Legionella strains form an essential part of outbreak investigations. DNA microarrays often comprise many DNA markers (features). Feature selection and the development of prediction models are particularly challenging in this domain with many variables and comparatively few subjects or data points. We aimed to compare modeling strategies to develop prediction models for classifying infections as clinical or environmental.

**Methods:**

We applied a bootstrap strategy for preselecting important features to a database containing 222 *Legionella pneumophila* strains with 448 continuous markers and a dichotomous outcome (clinical or environmental). Feature selection was done with 50 bootstrap samples resulting in a top 10 of most important features for each of four modeling techniques: classification and regression trees (CART), random forests (RF), support vector machines (SVM) and least absolute shrinkage and selection operator (LASSO). Validation was done in a second bootstrap re-sampling loop (200×) for evaluation of discriminatory model performance according to the AUC.

**Results:**

The top 5 of selected features differed considerably between the various modeling techniques, with only one common feature (“LePn.007B8”). The mean validated AUC-values of the SVM model and the CART model were 0.859 and 0.873 respectively. The LASSO and the RF model showed higher validated AUC-values (0.925 and 0.975 respectively).

**Conclusions:**

In the domain of *Legionella pneumophila*, which comprises many potential features for classifying of infections as clinical or environmental, the RF and LASSO techniques provide good prediction models. The identification of potentially biologically relevant features is highly dependent on the technique used, and should hence be interpreted with caution.

**Electronic supplementary material:**

The online version of this article (doi:10.1186/s13104-016-1945-2) contains supplementary material, which is available to authorized users.

## Background

The bacterium *Legionella pneumophila,* the causative agent for Legionnaires’ disease, is omnipresent in both natural and man-made aquatic environments. The major route of transmission is inhalation of the bacterium, which is spread into the air as an aerosol from its reservoir [1]. Genetic comparisons of clinical and environmental Legionella strains form an essential part of outbreak investigations [2, 3]. Such investigations previously showed that the distribution of genotypes within clinical strains significantly differed from the distribution in environmental strains [4–6].

To develop reliable statistical models for the discrimination between clinical and environmental strains, modeling techniques are required which can stabilize the feature selection. DNA microarrays may comprise thousands of DNA markers (features, *p*) and only a few hundred or even only a few dozen subjects (*n*; the “p > n” problem) [7].

Common statistical approaches for selecting features include filter methods, wrapper methods and embedded methods. Filter methods preselect features using a univariate technique with respect to the outcome (*T* test, Mann–Whitney-test, Pearson correlation coefficients), without being tuned to a specific type of modeling technique. By contrast, wrapper methods use a specific modeling technique to select features, and subsequently each selected feature set is used to train a model built with that same modeling technique; the performance of the model is usually tested on a hold-out set, resulting in a score for a specific feature set. Embedded methods are a catch-all group of techniques that perform feature selection as part of the model construction process [8, 9].

Popular feature selection methods nowadays are the least absolute shrinkage and selection operator method (LASSO) [10], recursive feature elimination, which is commonly used with support vector machines (SVM RFE) [11], and a backward feature selection method based on random forests (VARSEL RF) [12]. For stabilizing the feature selection, several authors proposed the use of ensemble feature selection based on bootstrap samples [13–15], a widely used technique in prediction research [16]. Several authors discussed double bootstrap or nested bootstrap procedures for both feature selection and performance estimation [17–22].

The aim of the present study was to compare statistical models that can be used to discriminate between clinical and environmental strains using a small number of features. We compared modeling techniques for developing prediction models with relevant genomic features related to pathogenicity. We focused on four modeling techniques: classification and regression trees (CART) [23], random forests (RF) [24], support vector machines (SVM) [25] and least absolute shrinkage and selection operator (LASSO) [26]. We used a nested bootstrap procedure, one for feature selection and one for predictive performance validation for a fair evaluation of a prediction model based on a relatively small data set.

## Methods

### Data

We analyzed the database of the Dutch National Legionella Outbreak Detection Programme as used before [27]. The database contained 222 *Legionella pneumophila* strains with 448 continuous markers and a dichotomous outcome. Of these strains, 49 were patient-derived strains from notified cases in the Netherlands in the period 2002–2006, and 173 were environmental strains that were collected during the source investigation for those patients. The 448 continuous markers were coded as LePn.###L## (e.g. LePn.032E12). The data were collected prospectively and anonymously. According to Dutch regulations, neither medical nor ethical approval was required to conduct the study, as no medical interventions were initiated and the study had no influence on medical care nor on decision making.

### Modeling techniques

We evaluated the modeling techniques CART, RF, SVM and LASSO, which are described below.

#### Classification and regression trees (CART)

The CART model is a tree-based classification and prediction model that uses recursive partitioning to split the training records into segments with similar output variable values. The modelling starts by examining the input variables to find the best split, measured by the reduction in an impurity index that results from the split. The split defines two subgroups, each of which is subsequently split into two further subgroups and so on, until the stopping criterion is met [23].

#### Random forest (RF)

Random forest is an ensemble classifier that consists of many decision trees and outputs the class that is the mode of the classes output by individual trees [24].

Each tree is constructed using the following algorithm:

1. Let the number of training cases be N, and the number of variables in the classifier be M.
2. We are told the number m of input variables to be used to determine the decision at a node of the tree; m should be much lower than M.
3. Choose a training set for this tree by choosing n times with replacement from all N available training cases (i.e. take a bootstrap sample). Use the rest of the cases to estimate the error of the tree, by predicting their classes.
4. For each node of the tree, randomly choose m variables on which to base the decision at that node. Calculate the best split based on these m variables in the training set.
5. Each tree is fully grown and not pruned (as may be done in constructing a normal tree classifier).

For prediction a new sample is pushed down the tree. It is assigned the label of the training sample in the terminal node it ends up in. This procedure is iterated over all trees in the ensemble, and the mode of the votes over all trees is used as the random forest prediction.

#### Support vector machine (SVM)

A support vector machine performs classification tasks by constructing hyperplanes in a multidimensional space that separate cases from non-cases. It claims to be a robust technique that maximizes the predictive accuracy of a model without overfitting the training data. SVM may particularly be suited to analyze data with large numbers of predictor variables. SVM has applications in many disciplines, including customer relationship management, image recognition, bioinformatics, text mining concept extraction, intrusion detection, protein structure prediction, and voice and speech recognition [25].

#### Least absolute shrinkage selection operator (LASSO)

Given a set of input measurements ${\text{x}}_{1} ,{\text{ x}}_{2} , \ldots ,{\text{ x}}_{\text{p}}$ and an outcome measurement y, the LASSO fits a linear model: ${\hat{\text{y}}} = {\text{b}}_{0} + {\text{b}}_{1} \times {\text{x}}_{1} + {\text{b}}_{2} \times {\text{x}}_{2} + \cdots + {\text{b}}_{\text{p}} \times {\text{x}}_{{{\text{p}} .}}$

It uses the following criterion: Minimize sum((y−ŷ)^2^) subject to sum(|b_j_|) ≤ s.

The first sum is taken over the cases in the dataset. The bound “s” is a tuning parameter. If “s” is large, the constraint has no effect and the solution is just the usual maximum likelihood regression of y on ${\text{B}}_{\text{i}} \left( {{\text{B}}_{1} , \ldots ,{\text{B}}_{50} } \right)$. For smaller values of s (s ≥ 0) the regression coefficients are shrunken versions of the maximum likelihood estimates. Often, some of the coefficients b_j_ are shrunk to zero. We used cross-validation to estimate the best value for “s” [26], and a logistic link function rather than linear regression.

### Reference techniques

As reference points for this evaluation, we applied the commonly used techniques VARSEL RF and SVM RFE to our database, which are examples of embedded methods. VARSEL RF is a feature selection technique based on random forests with backward stepwise elimination of features that are not important. SVM RFE is a recursive feature elimination technique. It is based on support vector machines, which eliminate feature redundancy resulting in compact feature sets.

### Model performance

We evaluated the stability of the feature selection and the validated performance by means of bootstrap re-sampling from the original database. The performance of a model resulting from a modeling technique was assessed using the area under the Receiver Operator Curve (AUC).

### Modeling strategy

**Fig. 1:**
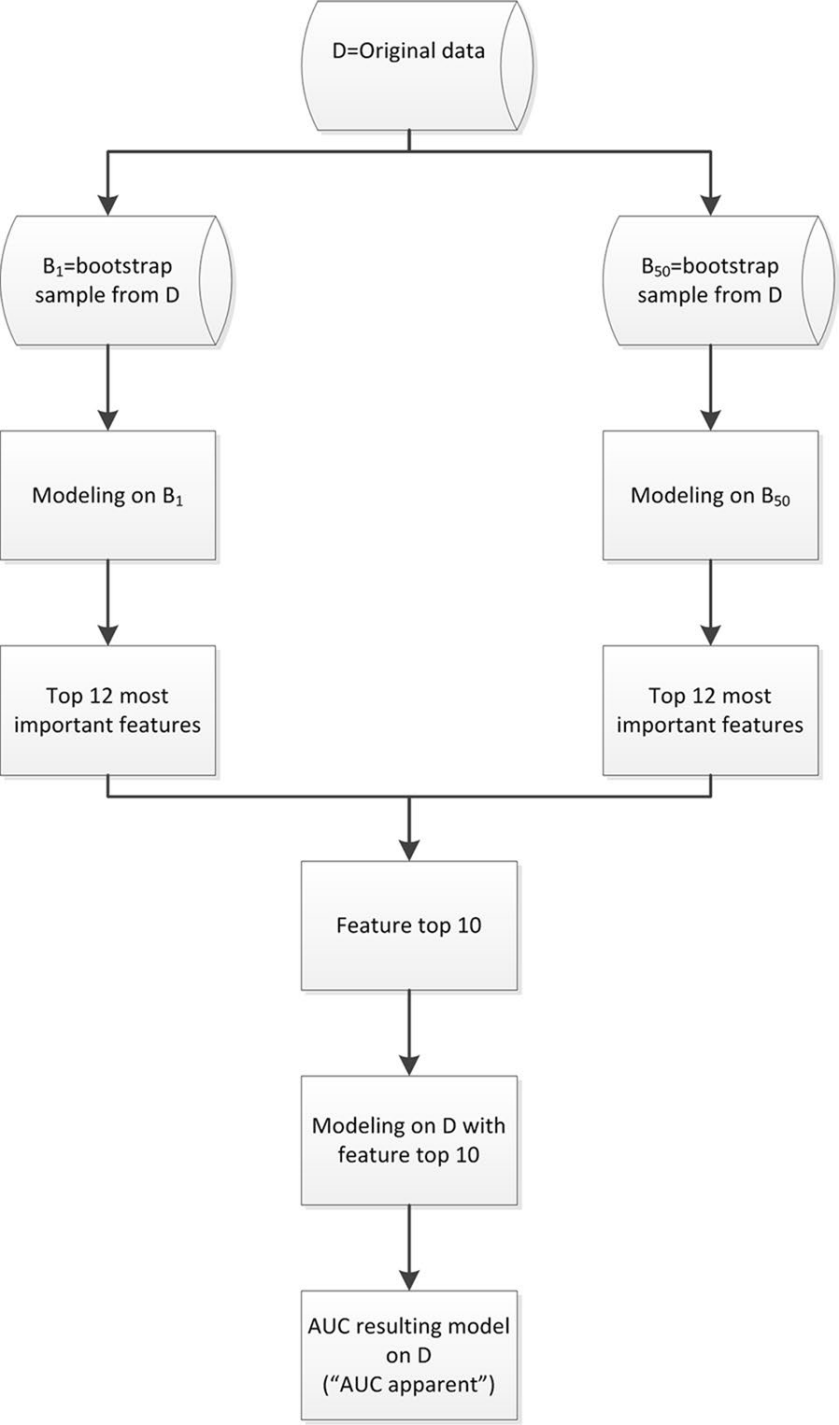
Feature selection and model development strategy

For a specific modeling technique, feature selection was done by bootstrap re-sampling from the original database D. We re-sampled 50 bootstraps ${\text{B}}_{\text{i}} \left( {{\text{B}}_{1} , \ldots ,{\text{B}}_{50} } \right)$ from the original database D. We applied the specific modeling technique on each B_i_ and determined for each B_i_ the top 12 of most important features, leading to 50 × 12 = 600 important features. From these 600 features, the top 10 of features with the highest frequency was extracted. With this feature top 10, a model was developed on the original database D with the specific modeling technique. For the resulting model the performance for the original database D was calculated (“AUC apparent”, Fig. 1).

### Validation of the strategy

To validate our strategy for a specific modeling technique, we performed a bootstrap procedure. We re-sampled a bootstrap sample B_j_ from the original data base D and from this bootstrap sample B_j_, we re-sampled 50 independent bootstraps ${\text{B}}_{\text{ji}} \left( {{\text{B}}_{\text{j1}} , \ldots ,{\text{B}}_{\text{j50}} } \right).$

**Fig. 2:**
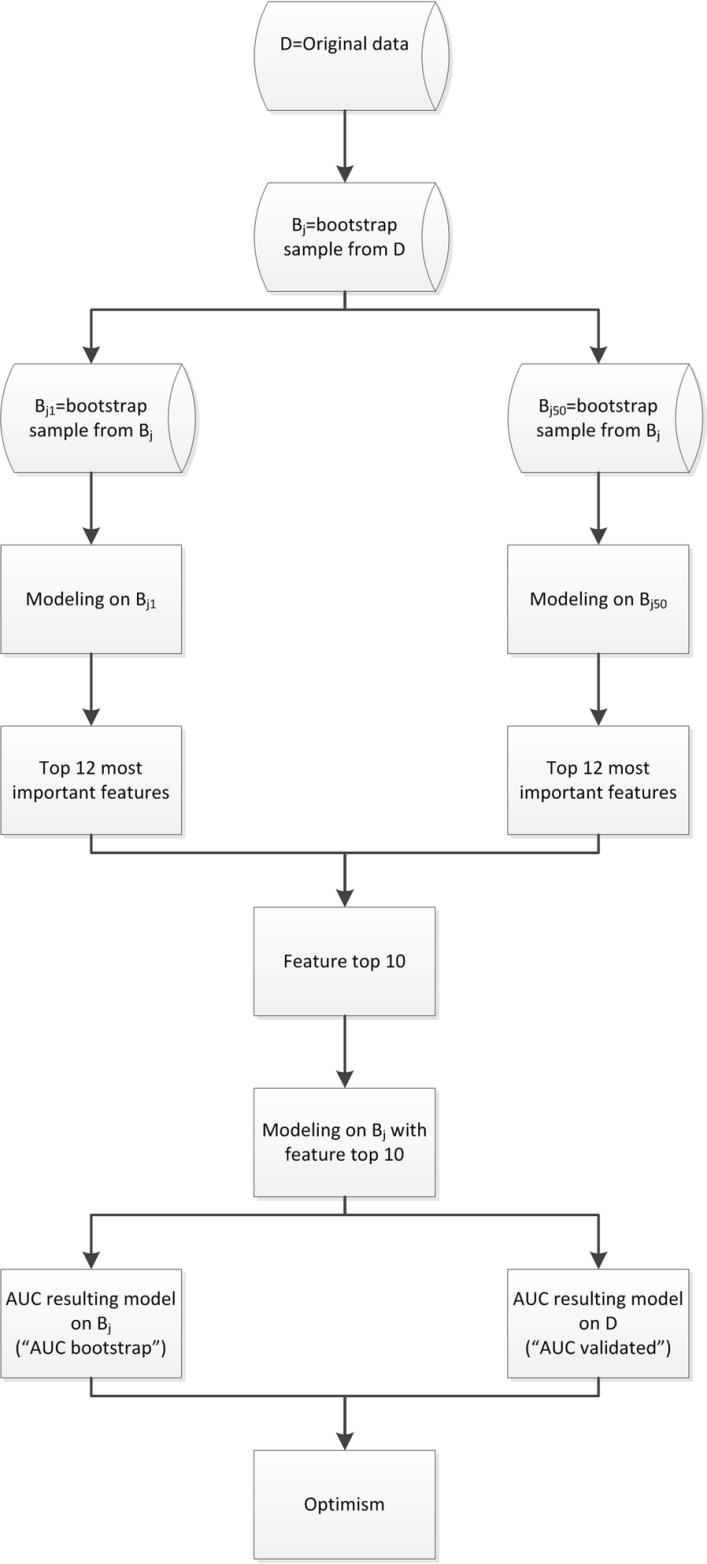
Evaluation of optimism for each strategy

We applied the specific modeling technique on each B_ji_ and determined for each B_ji_ the top 12 of most important features, leading to 50 × 12 = 600 important features. From these 600 features, the top 10 of features with the highest frequency was extracted. With this top 10 features, a model was developed on bootstrap sample B_j_ with the specific modeling technique. For the resulting model, the performance for B_j_ and the performance for the original data base D were calculated (“AUC bootstrap” and “AUC validated” respectively). The optimism of the resulting model was calculated as “AUC bootstrap” minus “AUC validated”. This process was repeated 200 times (B_1_ to B_200_, Fig. 2).

### Analysis

For the modeling and the analysis of these techniques, we used R 2.14, using default settings as far as possible. We used the libraries randomForest, caTools, rpart, caret, e1071, varSelRF and glmnet [28].

## Results

### Reference techniques

Feature selection with the reference techniques VARSEL RF and SVM RFE resulted in two different sets of features, only with LePn.007B8 as the common feature in the top 5 (Table 1). For the full list of features for each technique and for each bootstrap sample, we refer to Additional files 1 and 2. The mean validated AUC values of the models generated by these two techniques were 0.966 for VARSEL RF and 0.915 for SVM RFE (Table 2).

**Table 1:** Top 5 features VARSEL RF and SVM RFE and frequency of selection in 200 bootstrap resamples

Technique	Top 5 features and frequencies **[]**									
VARSELRF	LePn.007B8	[196]	LePn.032E12	[93]	LePn.004E8	[71]	LePn.015B2	[40]	LePn.035C6	[40]
SVMRFE	LePn.007B8	[88]	LePn.016E4	[80]	LePn.033H2	[77]	LePn.005H6	[60]	LePn.033D7	[54]

**Table 2:** Mean AUC and mean optimism VARSEL RF and SVM RFE

Technique	Apparent AUC	Bootstrap AUC		Validated AUC		Optimism	
		Mean	95 % CI	Mean	95 % CI	Mean	95 % CI
VARSELRF	0.904	0.966	[0.963; 0.969]	0.966	[0.963; 0.969]	0.000	[−0.004; 0.004]
SVMRFE	0.964	0.991	[0.990; 0.992]	0.915	[0.911; 0.919]	0.076	[0.072; 0.080]

### Other techniques

The top 5 of selected features differed among the other modeling techniques (CART, RF, SVM, LASSO). The only common feature in the top 5 of all four modeling techniques was feature LePn.007B8. Feature selection with RF resulted in four matches with feature selection based on VARSEL RF, and feature selection with LASSO resulted in three matches with feature selection with SVM RFE (Table 3). The selected features also differed within the various modeling techniques. For the full list of selected features for each technique and for each bootstrap sample, we refer to Additional files 3, 4, 5 and 6. The RF model showed the highest mean validated AUC value (0.975) followed by the LASSO model (0.925). The mean validated AUC values of the CART and the SVM models were 0.873 and 0.859 respectively (Table 4). The RF model showed a relatively low statistical optimism (0.005). Modeling with CART, SVM and LASSO resulted in prediction models with higher optimism (decrease in performance 0.064, 0.066 and 0.056 respectively, Table 4).

**Table 3:** Top 5 features CART, RF, SVM and LASSO and frequency of selection in 200 bootstrap resamples

Technique	Top 5 features and frequencies []									
CART	LePn.007B8	[200]	LePn.026A7	[93]	LePn.027A12	[76]	LePn.028A11	[71]	LePn.016E4	[66]
RF	LePn.007B8	[200]	LePn.032E12	[168]	LePn.004E8	[151]	LePn.035C6	[141]	LePn.016E4	[100]
SVM	LePn.007B8	[144]	LePn.035G3	[111]	LePn.009C5	[105]	LePn.012C5	[97]	LePn.024C3	[89]
LASSO	LePn.007B8	[187]	LePn.033H2	[146]	LePn.016E4	[131]	LePn.010B12	[83]	LePn.011B3	[77]

**Table 4:** Mean AUC and mean optimism CART, RF, SVM and LASSO

Technique	Apparent AUC	Bootstrap AUC		Validated AUC		Optimism	
		Mean	95 % CI	Mean	95 % CI	Mean	95 % CI
CART	0.929	0.937	[0.933; 0.942]	0.873	[0.868; 0.878]	0.064	[0.060; 0.068]
RF	0.938	0.980	[0.978; 0.981]	0.975	[0.973; 0.976]	0.005	[0.003; 0.008]
SVM	0.887	0.924	[0.918; 0.930]	0.859	[0.852; 0.866]	0.066	[0.061; 0.071]
LASSO	0.965	0.981	[0.980; 0.983]	0.925	[0.922; 0.928]	0.056	[0.053; 0.060]

## Discussion

Using a feature selection and validation strategy based on bootstrap procedures, we found that RF and LASSO modeling resulted in prediction models with high performance. The statistical optimism of the RF model was relatively low (0.005). By contrast, modeling with CART, SVM and LASSO resulted in prediction models which had a good validated performance, but with higher optimism in the apparent performance estimates (0.064, 0.066 and 0.056 respectively).

We applied two commonly used techniques as references: variable selection from random forests using backward variable elimination (VARSEL RF) and support vector machines using recursive feature elimination (SVM RFE). We applied these techniques to the same database and validated the resulting models by means of bootstrap re-sampling. These analyses showed that VARSEL RF had a high validated performance (AUC 0.966), whereas modeling with SVM RFE resulted in a validated performance of 0.915 and an optimism of 0.076.

We used the bootstrap procedure as described by Efron [16]. The original data set comprised 222 Legionella strains. Bootstrapping from that data set leads to 222 Legionella strains again in each bootstrap sample because it is based on simple re-sampling with replacement. We note than the 0.632+ variant of the standard bootstrap validation procedure uses only cases not used at model development. Empirical evaluations for binary prediction showed no advantage of this bootstrapping variant [29]. Hence, we did not use this approach in the estimation of the optimism of the models and the stability of the feature set.

Our results are in line with earlier findings, which showed that RF and LASSO are suitable modeling techniques for feature selection and that the resulting models have a good predictive performance [10, 11]. Our results with SVM modeling are in line with the work of Guyon et al. who suggested SVM RFE for feature selection [11]. However, the features selected with SVM and bootstrapping differed from the features selected with the SVM RFE approach. The validated predictive performance of our strategy with SVM modeling was inferior to the validated predictive performance with the SVM RFE approach (mean validated AUC 0.859 and 0.915 respectively).

We found that feature selection by means of VARSEL RF resulted in models with a high validated performance. This is in line with the findings of earlier studies that used a simpler validation procedure [27]. Likewise, RF modeling resulted in models with a very high performance (mean validated AUC 0.975). Feature selection with either of the two RF approaches resulted in four matching features (LePn.007B8, LePn.004E8, LePn.032E12 and LePn.035C6).

Feature selection with LASSO modeling resulted in a top 3 that was identical to the top 3 based on feature selection with SVM RFE. The relevance of this match is reinforced by the fact that feature selection with both these techniques resulted in models with a fairly high performance (validated AUC 0.915 and 0.925 respectively).

One of the limitations of our study is that we used one single database with features of a specific bacterium to compare the performance of the various modeling techniques. Future research should apply strong validation methods, such as our double bootstrap method, when analyzing comparable databases, such as databases comprising Legionella strains from other countries. An even stronger validation would be achieved by testing the models on new, independent data. Another limitation is that we restricted our research to four modeling techniques (CART, RF, SVM and LASSO). Various other techniques might also be suitable for feature selection and prediction in a domain with many variables and few subjects.

## Conclusions

In the domain of *Legionella pneumophila*, which comprises many potential features for classifying of infections as clinical or environmental, the RF and LASSO techniques provide good prediction models. The identification of potentially biologically relevant features is highly dependent on the technique used, and should hence be interpreted with caution.

## Additional files

10.1186/s13104-016-1945-2 Feature set VARSEL RF.
10.1186/s13104-016-1945-2 Feature set SVM RFE.
10.1186/s13104-016-1945-2 Feature set CART.
10.1186/s13104-016-1945-2 Feature set RF.
10.1186/s13104-016-1945-2 Feature set SVM.
10.1186/s13104-016-1945-2 Feature set LASSO.

## Abbreviations

DNA: deoxyribonucleic acid
LASSO: least absolute shrinkage and selection operator
SVM RFE: support vector machines recursive feature elimination
VARSEL RF: variable selection random forest
CART: classification and regression trees
RF: random forests
SVM: support vector machines
